# Pharmacological Properties, Volatile Organic Compounds, and Genome Sequences of Bacterial Endophytes from the Mangrove Plant *Rhizophora apiculata* Blume

**DOI:** 10.3390/antibiotics10121491

**Published:** 2021-12-05

**Authors:** Ton That Huu Dat, Phung Thi Thuy Oanh, Le Canh Viet Cuong, Le Tuan Anh, Le Thi Hong Minh, Hoang Ha, Le Tung Lam, Pham Viet Cuong, Hoang Le Tuan Anh

**Affiliations:** 1 Mientrung Institute for Scientific Research, Vietnam National Museum of Nature, Vietnam Academy of Science and Technology, 321 Huynh Thuc Khang, Hue City 49117, Vietnam; oanh5794@vnmn.vast.vn (P.T.T.O.); lcvcuong@vnmn.vast.vn (L.C.V.C.); ltanh@vnmn.vast.vn (L.T.A.); 2 Institute of Marine Biochemistry, Vietnam Academy of Science and Technology, 18 Hoang Quoc Viet, Cau Giay, Hanoi 10072, Vietnam; lhminh@imbc.vast.vn; 3 Institute of Biotechnology, Vietnam Academy of Science and Technology, 18 Hoang Quoc Viet, Cau Giay, Hanoi 10072, Vietnam; hoanghapcb@ibt.ac.vn (H.H.); letunglam@ibt.ac.vn (L.T.L.); 4 Center for Research and Technology Transfer, Vietnam Academy of Science and Technology, 18 Hoang Quoc Viet, Cau Giay, Hanoi 10072, Vietnam

**Keywords:** biological activity, biosynthesis gene clusters, endophytic bacteria, GC-MS, genome, *Rhizophora apiculata*

## Abstract

Mangrove plant endophytic bacteria are prolific sources of bioactive secondary metabolites. In the present study, twenty-three endophytic bacteria were isolated from the fresh roots of the mangrove plant *Rhizophora apiculata*. The identification of isolates by 16S rRNA gene sequences revealed that the isolated endophytic bacteria belonged to nine genera, including *Streptomyces*, *Bacillus*, *Pseudovibrio*, *Microbacterium*, *Brevibacterium*, *Microbulbifer*, *Micrococcus*, *Rossellomorea*, and *Paracoccus*. The ethyl acetate extracts of the endophytic bacteria’s pharmacological properties were evaluated in vitro, including antimicrobial, antioxidant, α-amylase and α-glucosidase inhibitory, xanthine oxidase inhibitory, and cytotoxic activities. Gas chromatography–mass spectrometry (GC-MS) analyses of three high bioactive strains *Bacillus* sp. RAR_GA_16, *Rossellomorea vietnamensis* RAR_WA_32, and *Bacillus* sp. RAR_M1_44 identified major volatile organic compounds (VOCs) in their ethyl acetate extracts. Genome analyses identified biosynthesis gene clusters (BGCs) of secondary metabolites of the bacterial endophytes. The obtained results reveal that the endophytic bacteria from *R. apiculata* may be a potential source of pharmacological secondary metabolites, and further investigations of the high bioactive strains—such as fermentation and isolation of pure bioactive compounds, and heterologous expression of novel BGCs in appropriate expression hosts—may allow exploring and exploiting the promising bioactive compounds for future drug development.

## 1. Introduction

Plant endophytic bacteria are live and thrive inside plants without causing harmful effects to their host plants [1]. The endophytic bacteria provide numerous benefits to their host plants, including promoting the growth of host plants, enhancing the resistance of host plants against diseases, and increasing the tolerance of host plants to stressful environmental conditions [2,3,4]. Endophytic bacteria have been found in different parts of plants, such as roots, stems, leaves, seeds, fruits, tubers, ovules and nodules; however, roots contain the highest number of bacterial endophytes when compared to other plant parts [4,5].

The mangrove ecosystem is characterized by highly changeable environmental conditions such as salinity, temperature, nutrients, and tidal currents, making it one of the most productive ecosystems [6]. Mangrove plants growing under such frequent and sporadic environmental changes provide special ecological niches for diverse endophytic microbial communities [7]. In addition, the variable environmental conditions in mangrove ecosystems may serve as excellent selectors for structurally distinct secondary metabolites with intriguing pharmacological effects. Along with their host plants, endophytic bacteria have also been reported to be a rich source of secondary metabolites with promising pharmacological properties [8,9,10,11]. Of these, many compounds exhibit potent biological activities and may be considered as lead compounds for further drug developments.

*Rhizophora apiculata* is a “true” mangrove plant belonging to the genus *Rhizophora*, of the family Rhizophoraceae. The genus *Rhizophora* is generally found in intertidal zones and is distributed in both the Indo–West Pacific and Atlantic East Pacific regions [12]. Phytochemical investigations on *R. apiculata* reveal its structurally diverse secondary metabolites, including alkaloids, terpenoids, flavonoids, aliphatic alcohols, and phenolic derivatives [13,14,15,16,17]. The isolated compounds from this species have been reported to exhibit promising pharmacological properties, such as antimicrobial, antiviral, antioxidant, antidiabetic, anticancer, and hepatoprotective activities [15,18,19,20,21,22,23]. Apart from *R. apiculata*, its endophytic microorganisms produce various secondary metabolites with valuable biological properties such as antimicrobial, antiviral, and anticancer activities [11,24,25,26]. 

**Figure 1:**
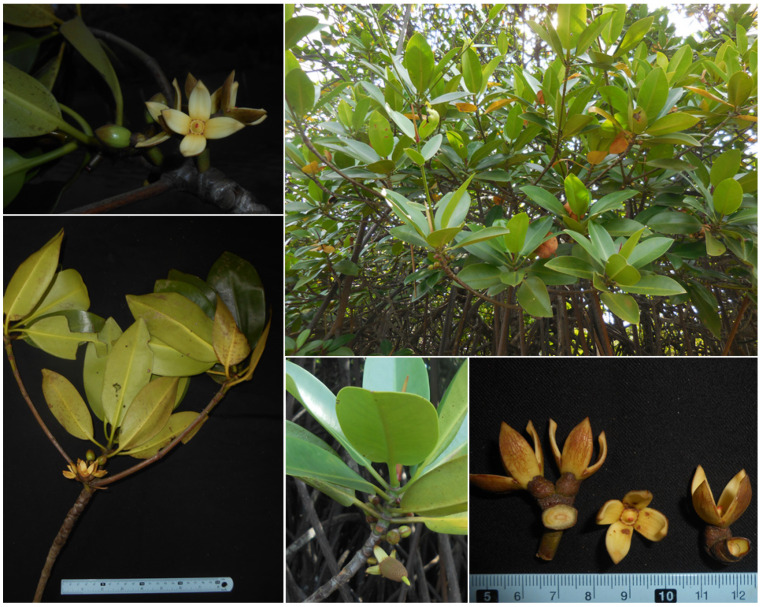
*Rhizophora apiculata* collected from the Bu Lu mangrove forest, Phu Loc district, Thua Thien Hue province, Vietnam.

With long coastlines up to 3260 km, coastal mangrove forests are among the most important ecosystems in Vietnam. The Vietnamese mangrove forests cover an area of 155,290 ha with 109 mangrove plant species. “True” mangrove plants consist of 37 species belonging to 20 genera of 14 families, whereas “associate” mangrove plants consist of 72 species belonging to 36 genera of 28 families. Among them, the most dominant mangrove plants are species of the genus *Rhizophora* [27]. In the present study, we isolated endophytic bacteria from the fresh roots of *R. apiculata* collected in the mangrove forest of Bu Lu, Phu Loc district, Thua Thien Hue province, Vietnam (Figure 1), and evaluated their potential pharmacological properties. The composition of volatile organic compounds present in the high bioactive extracts was determined by gas chromatography–mass spectrometry (GC-MS) analyses. Additionally, the biosynthesis gene clusters of secondary metabolites were discovered by the genome analysis. The obtained results in the present study reveal that the endophytic bacteria from *Rhizophora apiculata* are potential sources of biological compounds and further investigations, e.g., isolation of active pure compounds and heterologous expression of novel biosynthesis gene clusters of secondary metabolites should be performed in order to explore and exploit the novel bioactive compounds for drug development.

## 2. Results

### 2.1. Isolation and Identification of Endophytic Bacteria

**Figure 2:**
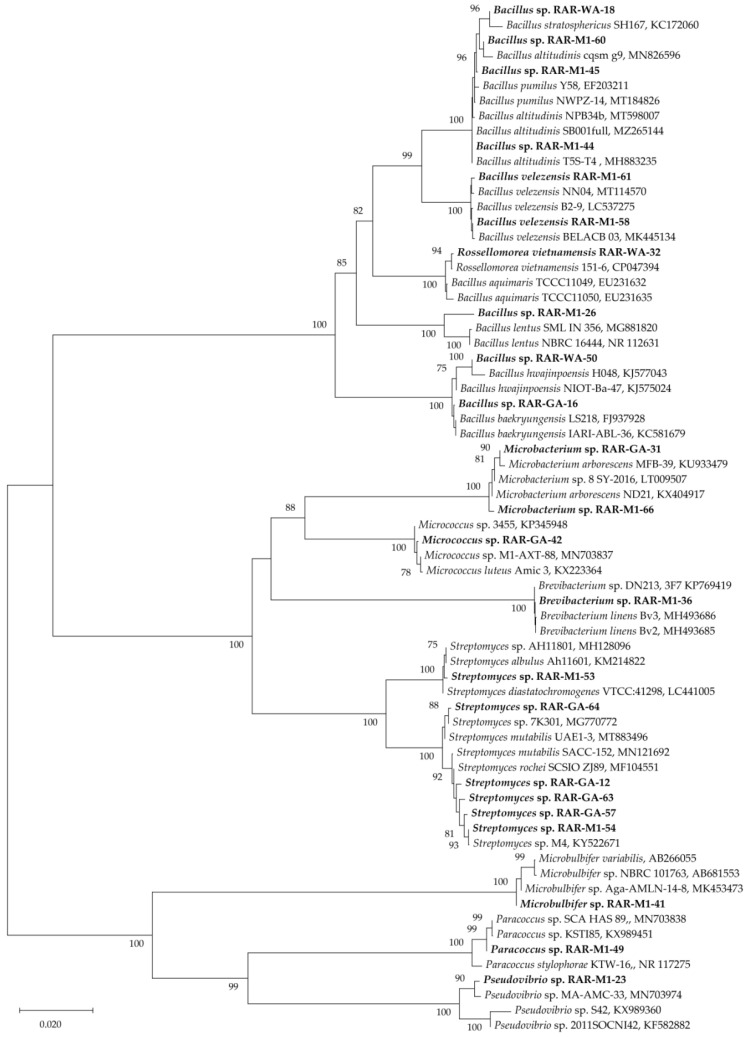
Phylogeny of 16S rRNA gene sequences of the endophytic bacteria in our study (bold letters) and from the National Center for Biotechnology Information (NCBI) GenBank. Bootstrap support values of branches greater than 75% are given above the corresponding branches.

Twenty-three endophytic bacterial strains were isolated from the fresh roots of the mangrove plant Rhizophora apiculata. Based on their 16S rRNA gene sequences, the bacterial strains were identified as belonging to nine different genera, i.e., *Streptomyces*, *Bacillus*, *Pseudovibrio*, *Microbacterium*,* Rossellomorea*, *Brevibacterium*, *Microbulbifer,* *Micrococcus*, and *Paracoccus* (Table 1). The phylogenic tree based on their 16S rRNA gene sequences is shown in Figure 2. All isolated strains were cultured in nutrient broth and their ethyl acetate extracts were prepared for evaluating their potential pharmacological properties.

### 2.2. Pharmacological Evaluation of the Ethyl Acetate Extracts

#### 2.2.1. Antimicrobial Activity

The antimicrobial assays showed that 18 of 23 bacterial extracts exhibited antimicrobial activity against at least one of the tested microbes with minimum inhibitory concentration (MIC) values from 16–256 µg/mL (Table 2). Among them, nine extracts exhibited antimicrobial activity against *S. aureus*, eleven extracts exhibited antimicrobial activity against *E. faecalis*, eight extracts exhibited antimicrobial activity against *E. coli*, eight extracts exhibited antimicrobial activity against *P. aegurinosa*, and ten extracts exhibited antimicrobial activity against *C. albicans*. Several extracts showed antimicrobial activity against multiple tested microbes, for example, three extracts (RAR_GA_16, RAR_WA_18, RAR_M1_44) exhibited antimicrobial activity against four tested microbes, eight extracts (RAR_GA_12, RAR_WA_32, RAR_M1_36, RAR_M1_45, RAR_WA_50, RAR_GA_57, RAR_M1_60, RAR_M1_66) exhibited antimicrobial activity against three tested microbes, three extracts (RAR_GA_31, RAR_M1_53, RAR_GA_63) exhibited antimicrobial activity against two tested microbes, and four extracts (RAR_M1_23, RAR_M1_54, RAR_M1_58, RAR_GA_64) exhibited antimicrobial activity against one tested microbe. Notably, several extracts showed significant antimicrobial activity with MIC values between 16 and 64 µg/mL.

#### 2.2.2. Antioxidant Activity

The antioxidant activity of the bacterial extracts was evaluated by their DPPH (1,1-diphenyl-2-picrylhydrazyl) and ABTS (2,2-azino-bis-3-ethylbenzothiazoline-6-sulfonic acid) radical scavenging effects (Table 3). Antioxidant assays showed that nine extracts exhibited significant DPPH radical scavenging activity with IC_50_ values from 43.52 ± 3.87 to 88.32 ± 4.13 µg/mL and eleven extracts exhibited significant ABTS radical scavenging activity with IC_50_ values from 56.29 ± 4.61 to 81.45 ± 3.64 µg/mL. Among them, eight extracts, i.e., RAR_GA_16, RAR_M1_23, RAR_GA_31, RAR_WA_32, RAR_GA_42, RAR_M1_44, RAR_M1_58, and RAR_M1_61 exhibited both DPPH and ABTS radical scavenging activities.

#### 2.2.3. α-Amylase, α-Glucosidase, and Xanthine Oxidase Inhibitory Activities

The inhibitory effects of the bacterial extracts against several enzymes related to diabetes and gout, i.e., α-amylase, α-glucosidase, and xanthine oxidase, also were evaluated (Table 4). Bioassays showed that eleven extracts exhibited significant α-amylase inhibitory activity with IC_50_ values from 33.51 ± 4.62 to 131.36 ± 5.41 µg/mL, and nine extracts exhibited significant α-glucosidase inhibitory activity with IC_50_ values from 53.68 ± 3.12 to 193.44 ± 6.73 µg/mL. Among them, six extracts exhibited both α-amylase and α-glucosidase inhibitory activities, such as RAR_GA_16, RAR_GA_31, RAR_WA_32, RAR_M1_44, RAR_M1_49, and RAR_GA_57. Notably, several extracts exhibited better α-amylase and α-glucosidase inhibitory activities than the positive control acarbose (IC_50_ = 89.34 ± 3.61 and 217.46 ± 6.38 for α-amylase and α-glucosidase, respectively). Regarding the XO enzyme, ten extracts exhibited significant XO inhibitory activity with IC_50_ values from 54.57 ± 2.53 to 94.36 ± 4.74 µg/mL.

#### 2.2.4. Cytotoxic Activity

The cytotoxic activity of the bacterial extracts against three cancer cell lines, i.e., MCF-7 (human breast carcinoma), A549 (human lung carcinoma), and HeLa (human cervix carcinoma), are shown in Table 5. Cytotoxic bioassays showed that six extracts exhibited significant cytotoxic activity against the cell line MCF-7 with IC_50_ values from 36.48 ± 2.63 to 83.24 ± 4.51 µg/mL, four extracts exhibited significant cytotoxic activity against the cell line A549 with IC_50_ values from 21.52 ± 3.22 to 89.53 ± 5.31 µg/mL, and three extracts exhibited significant cytotoxic activity against the cell line A549 with IC_50_ values from 41.27 ± 3.42 to 97.53 ± 5.31 µg/mL. Of these, two extracts exhibited cytotoxic activity against two cell lines, i.e., RAR_WA_32 and RAR_WA_50, and two extracts exhibited cytotoxic activity against all three tested cell lines, i.e., RAR_GA_16 and RAR_M1_44.

### 2.3. Volatile Chemical Composition of the Endophytic Bacterial Extracts by GC-MS

The bioassays revealed that the ethyl acetate extracts of the three endophytic strains *Bacillus* sp. RAR_GA_16, *Rossellomorea vietnamensis* RAR_WA_32, and *Bacillus* sp. RAR_M1_44 displayed the most potential pharmacological properties. Thus, the composition of volatile organic compounds present in their ethyl acetate extracts was investigated by GC-MS analyses. The GC-MS analysis identified 29, 78, and 42 peaks of VOCs from the extract of the bacterial strains RAR_GA_16, RAR_WA_32, and RAR_M1_44, respectively (Supplementary Tables S1–S3). By comparing with the spectra databases of the known compounds, 7 compounds were identified in the extract of *Bacillus* sp. RAR_GA_16 (Table 6) including hexahydro-pyrrolo[1,2-a]pyrazine-1,4-dione (13.15%), 3-isobutylhexahydropyrrolo[1,2-a]pyrazine-1,4-dione (8.15%), palmitic acid (2.09%), diisooctyl phthalate (6.90%), linoleic acid (1.57%), 9(E)-octadecenoic acid (9.17%), and stearic acid (3.02%), 26 compounds were identified from the extract of *Rossellomorea vietnamensis *RAR_WA_32 with the major components including dioctyl phthalate (58.09%), palmitic acid (5.56%), 3-isobutylhexahydropyrrolo[1,2-a]pyrazine-1,4-dione (4.56%) stearic acid (3.55%), benzeneacetic acid (1.92%), and 3-benzyl-hexahydro-pyrrolo[1,2-a]pyrazine-1,4-dione (1.875), and 6 compounds were identified from the extract of *Bacillus* sp. RAR_M1_44 including palmitic acid (0.51%), (Z,Z)-9,12-octadecadienoic acid (0.33%), 6-octadecenoic acid (1.93%), stearic acid (0.34%), and 1,2-benzenedicarboxylic acid (0.99%) (Table 6). Interestingly, several compounds were found in the extracts of all three bacterial strains *Bacillus* sp. RAR_GA_16, *Rossellomorea vietnamensis* RAR_WA_32, and *Bacillus* sp. RAR_M1_44 (i.e., palmitic acid and stearic acid); however, the quantities of the compounds varied among the extracts.

### 2.4. Genome Sequencing, Assembly, and Annotation of Biosynthesis Gene Clusters of Secondary Metabolites

In order to discover the biosynthesis gene clusters (BGCs) of secondary metabolites of the isolated bacteria, we sequenced their genomes and annotated BGCs of secondary metabolites from the genome data. The genomic features of three bacterial strains *Bacillus* sp. RAR_GA_16, *Rossellomorea vietnamensis* RAR_WA_32, and *Bacillus* sp. RAR_M1_44 are shown in Table 7. The size of the bacterial genomes was from 3,768,026 to 4,494,267 bp with GC contents from 40.69 to 44.09%. Assembly results showed that completion of the genomes was from 96.6 to 99.8%, with the maximum contig length from 770,470 to 2,585,281 bp and N50 contig length from 501,856 to 2,582,281 bp. Annotation of the genomes by Prokka showed that the genomes contain 3807–4610 coding sequences (CDSs), 22–27 rRNA, 81–111 tRNA, and 0–1 tmRNA.

The genomes were analyzed for the presence of secondary metabolite biosynthetic gene clusters (BGCs) using antiSMASH. Annotation results showed that the genome of *Bacillus* sp. RAR_GA_16 contains 22 BGCs, including clusters related to the biosynthesis of linear azol(in)e-containing peptide (LAP), bacteriocin, lassopeptide, siderophore, type III polyketide (T3PKS), terpene, and several unknown compounds. Several BGCs shared their identity to the biosynthesis clusters of known compounds in Minimum Information about a Biosynthetic Gene cluster (MIBiG) database, such as paeninodin, butirosin A, butirosin B, carotenoid, thaxteramide C, and fengycin with the identities of 4–80% (Table 8).

Regarding the genome of *Rossellomorea vietnamensis* RAR_WA_32, annotation results showed that the genome contains 16 BGCs, including clusters related to the biosynthesis of terpene, LAP, type III polyketide, fatty acid, other unspecified ribosomally synthesised and post-translationally modified peptide product (RiPP-like), and saccharide (likely from primary metabolism). Several BGCs shared their identity to known biosynthesis clusters in the MIBiG database, such as pyxidicycline A, pyxidicycline B, A40926, and carotenoid with the identities of 3–50% (Table 8).

**Figure 3:**
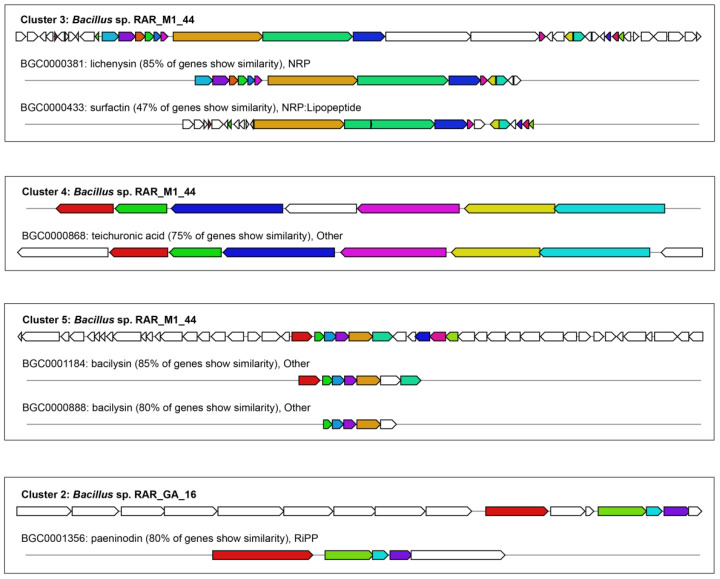
Similarity of predicted BGCs from genomes and the MIBiG database.

In the case of the genome of *Bacillus* sp. RAR_M1_44, it contains 16 BGCs, including clusters related to the biosynthesis of terpene, siderophore, betalactone, nonribosomal peptide (NRP), type III polyketide, bacteriocin, others and unknown compounds. Several BGCs shared their identity to the biosynthesis clusters of known compounds in the MIBiG database, such as carotenoid, fengycin, lichenysin, teichuronic acid, and bacilysin with the identities of 50–85% (Table 8). The gene structures of BCGs having the identities > 70% with known compound clusters in the MIBiG database are shown in Figure 3.

The BGCs predicted from the genomes were analyzed by the Biosynthetic Genes Similarity Clustering and Prospecting Engine (BIG-SCAPE) to group the homologous BGCs into gene cluster families (GCFs), which can be responsible for the production of the same compound or similar compounds. Interestingly, no GCF was found between the BCGs from the genomes as well as between the BCGs from the genomes and the BGCs from the MIBiG database.

The ketide synthase (KS) and condensation (C) domains are the most conserved catalytic domains of polyketide synthase (PKS) and non-ribosomal peptide synthetase (NRPS) genes, respectively. Therefore, in order to predict the secondary metabolites of polyketides and non-ribosomal peptides, KS- and C- domains from the genomes were analyzed by the Natural Product Domain Seeker (NapDos). Analyses showed that the genomes of *Rossellomorea vietnamensis* RAR_WA_32 and *Bacillus* sp. RAR_M1_44 contain only KS domains without C domains (Table 9). These KS sequences shared low identities with the KS sequences of the known products such as kirromycin, mycinamicin, rifamycin, amphotericin, spinosad, and dynemicin (26–45% identity), except for fatty acids with 71–76% identity. Regarding the genome of *Bacillus* sp. RAR_GA_16, three KS domains and eleven C domains were found in its genome (Table 9). These sequences shared low identities with the domain sequences of the known products such as mycocerosic acid, lychenicin, surfactin, and actinomycin (22–58%), except for fatty acid (84%).

## 3. Discussion

Mangrove plants harbour diverse endophytic bacteria and fungi. Reviews on mangrove plant-associated microbial secondary metabolites revealed that the secondary metabolites possess promising pharmacological properties [8,9,10,11]. Unsurprisingly, mangrove endophytic microbes have been attracting the considerable attention of pharmacological investigators because of their bioactive secondary metabolites, especially when their host plants are also used as native traditional/folk medicines [69]. Over the last decade, almost 1000 new natural products have been reported from mangrove associated microbes, of these, ~850 are derived from fungi and ~120 are derived from bacteria (the majority of them derived from endophytes) [10].

In the present study, twenty-three endophytic bacteria belonging to nine genera (i.e., *Microbulbifer*, *Streptomyces*, *Bacillus*, *Rossellomorea*, *Micrococcus*, *Paracoccus*, *Microbacterium*, *Pseudovibrio*, and *Brevibacterium*) were isolated from the fresh roots of *R. apiculata*. Previous investigations revealed that the genera *Bacillus*, *Streptomyces*, and *Pseudovibrio* have been isolated frequently from mangrove plants, whereas *Microbulbifer*, *Micrococcus*, *Paracoccus*, *Microbacterium*, and *Brevibacterium* have been isolated less frequently [10,70,71,72,73]. Genome analyses of the genera *Bacillus*, *Streptomyces*, and *Pseudovibrio* have reported that genomes of these genera contain versatile and diverse genes of metabolic pathways as well as genes linked to symbiosis and lifestyles, allowing for host switching and adapting to various environments [74,75,76,77,78,79,80].

The endophytic bacterial ethyl acetate extracts in the present study were evaluated for their potential pharmacological properties. The bioassays revealed that these endophytic bacteria may be promising sources of bioactive secondary metabolites because their extracts exhibited antimicrobial, cytotoxic, antioxidant, and enzyme inhibitory effects (i.e., α-amylase, α-glucosidase, and xanthine oxidase). These results are consistent with previous investigations of bioactive natural products from endophytic microorganisms with the mangrove plant *R. apiculata*. Rukachaisirikul et al. [81] isolated nineteen secondary compounds from the endophytic fungus *Acremonium* sp. PSU-MA70 from the plant *R. apiculata*. Among them, the two compounds 8-deoxytrichothecin and trichodermol exhibited antifungal activity against *C. albicans* and *Cryptococcus neoformanns* with MIC values of 16–64 μg/mL. Klaiklay et al. [82] reported the isolation of 7 antifungal compounds from the endophytic fungus *Pestalotiopsis* sp. PSU-MA69 from the plant *R. apiculata*, including pestalolide, (s)-penipratynolene, pestalotether A–B, pestheic acid, chloroisosulochrin dehydrate, and chloroisosulochrin, with MICs of 128–200 μg/mL. Zhou et al. [26] isolated three antimicrobial compounds, fusolanone A-B and fusaric acid, from the endophytic fungus *Fusarium solani* HDN15-410 from the plant *R. apiculata*. These compounds exhibited good antimicrobial activity against a wide range of pathogenic microbes, i.e., *P. aeruginosa*, *Monilia albican*, *B. subtilis*, and *Vibrio parahaemolyticus*, with MICs of 6.25–50 μg/mL. Fan et al. [24] isolated nine anti-H1N1 viral indole-diterpenoids from the fungus *Penicillium camemberti* OUCMDZ-1492 around roots of *R. apiculata* with IC_50_ values of 6.6–77.9 μM. Chaeprasert et al. [25] isolated 1921 fungal endophytic strains from 10 mangrove plant species *R. apiculata*, *R. mucronata*, *Ceriops decandra*, *Sonneratia alba*, *Lumnitzera littorea*, *Avicennia alba*, *Acanthus ilicifolius*, *Xylocarpus granatum*, *X. moluccensis*, and *Thespesia populneoides*. Among them, the ethyl acetate extract of 22 strains exhibited antimicrobial activity against *B. subtilis*, *E. coli*, *P. aeruginosa*, and *S. aureus* with zone inhibition diameters of 10*–*40 mm, and the ethyl acetate extract of 26 strains exhibited cytotoxic activity against the cancer cell lines A375 (malignant melanoma), HepG2 (liver hepatoblastoma), SW620 (colorectal adenocarcinoma), Jurkat (acute T cell leukemia), and KatoIII (gastric carcinoma). Most extracts inhibited the cancer cell lines with growth inhibition of >60%. Klaiklay et al. [83] isolated cytotoxic and antibacterial compounds from the endophytic fungus *Phomopsis* sp. PSU-MA214 from the plant *R. apiculata*. Compound (2R,3S)-7-ethyl-1,2,3,4-tetrahydro-2,3,8-trihydroxy-6-methoxy-3-methyl-9,10-anthracendione exhibited cytotoxicity against the cancer cell line MCF-7 with IC_50_ of 27 μg/mL and antibacterial activity against *S. aureus* and methicillin-resistant *S. aureus* with MICs of 128 μg/mL and 64 μg/mL, respectively, whereas compound phomonitroester exhibited cytotoxicity against the cancer cell line KB with IC_50_ of 43 μg/mL. Klaiklay et al. [84] isolated an antimicrobial compound, tremulenolide A, from the endophytic fungus *Flavodon flavus* PSU-MA201 from the plant *R. apiculata*. The compound exhibited antimicrobial activity against *S. aureus* and *C. neoformans* with MICs of 128 μg/mL.

The ethyl acetate extracts of three endophytic bacteria with the most potential biological properties, i.e., *Bacillus* sp. RAR_GA_16, *Rossellomorea vietnamensis* RAR_WA_32, and *Bacillus* sp. RAR_M1_44, were investigated for their volatile chemical components by GC-MS analyses. Interestingly, many compounds identified in the extracts of three endophytic bacteria have been reported to have biological activities (e.g., antibacterial, antifungal, antivirus, anticancer, anti-inflammatory, antioxidant, nematicidal, anti-quorum sensing, tyrosinase inhibitory, anti-biofilm, and anti-mutagenic activities) by previous studies (Table 6). These GC-MS analyses support the biological activity evaluation results of the ethyl acetate extracts. Additionally, it is noted that the identified VOCs from the extracts only accounted for 44.05%, 80.98%, and 4.10% of the total amount of VOCs in the extracts of *Bacillus* sp. RAR_GA_16, *Rossellomorea vietnamensis* RAR_WA_32, and *Bacillus* sp. RAR_M1_44, respectively, whereas many VOCs in the extracts were not identified by their low match quality with the spectra data of known compounds (Supplementary Tables S1–S3). This implies that many VOCs in the extracts may be undescribed or novel compounds.

In the present study, the genomes of three potential endophytic bacteria, i.e., *Bacillus* sp. RAR_GA_16, *Rossellomorea vietnamensis* RAR_WA_32, and *Bacillus* sp. RAR_M1_44, were also sequenced and the presence of BGCs of secondary metabolites in their genomes was discovered. Annotations using the antiSMASH found 16–22 BGCs, 3–5 KS domains, and 11 C domains in the bacterial genomes. However, the majority of BGCs, KS- and C- domains shared low identity with BGC, KS- and C- sequences in the known product database. Additionally, the BIG-SCAPE analysis also indicated that no BGCs predicted from the genomes were grouped in GCFs of known products in the MIBiG database. These findings suggest that the BGCs of the endophytic bacteria may biosynthesize novel compounds or compounds for which their BGCs have not been known. Interestingly, no GCF was found between the BGCs predicted from the genomes, implying that the BGCs of the endophytic bacteria may biosynthesize dissimilar compounds. The obtained results in the present study indicate that these endophytic bacteria may be potential sources for the discovery of novel bioactive metabolites, and further investigations, e.g., fermentation and isolation of pure bioactive compounds, as well as heterologous expression of novel BGCs in appropriate expression hosts, will allow exploring and exploiting novel bioactive compounds as well as demonstrating their biosynthesis pathways.

## 4. Materials and Methods

### 4.1. Plant Collection

The mangrove plant *Rhizophora apiculata* (Figure 1) was collected from the mangrove forest of Bu Lu, Phu Loc district, Thua Thien Hue province, Vietnam, in March 2019. The plant sample was transported to the laboratory of the Department of Biotechnology, Mientrung Institute for Scientific Research, for the isolation of the endophytic bacteria.

### 4.2. Isolation and Identification of the Endophytic Bacteria

The fresh plant root systems were washed thoroughly with running water to remove soil particles. The root surfaces were then washed in sterile distilled water six times, soaked in 70% ethanol for 5 min followed by 5% sodium hypochlorite for 10 min, and again in 70% ethanol for 60 s before washing in distilled water three times.

The root samples (5 g) were crushed in 5 mL of sterile distilled water. A total of 100 µL of root solution was spread on M1 medium (1.0% soluble starch, 0.4% yeast extract, 0.2% peptone, 1.5% agar), Gause no. 1 medium (2.0% soluble starch, 0.1% KNO_3_, 0.05% NaCl, 0.05% K_2_HPO_4_, 0.05% MgSO_4_, 0.001% FeSO_4_, 1.5% agar), and water agar (1.5% agar in mangrove water). The plates were incubated at 30 °C for 3–5 days. After incubation, colonies with different morphological characteristics (e.g., color, shape, and consistency) were streaked on nutrient agar plates (NA, Himedia, Mumbai, India) to obtain the pure isolates [70].

The representative isolates with different morphotypes were identified by colony PCR [85]. In brief, for cell lysis, the culture broths (2 mL) were centrifuged at 14,000× *g* for 10 min and the obtained pellets were suspended in 50 µL nuclease-free water. Subsequently, the cell suspension was stored at −20 °C for 2 h, followed by incubation at 98 °C for 10 min. The 16S rRNA gene of the isolates was directly amplified with universal primers 27f (5′-AGAGTTTGATCCTGGCTCAG-3′) and 1492r (5′-GGTTACCTTGTTACGACTT-3′) [86] with the following PCR program: an initial denaturation at 94 °C for 5 min, followed by 30 cycles of denaturation at 94 °C for 1 min, annealing at 56 °C for 50 s, amplification at 72 °C for 1.5 min, and a final extension at 72 °C for 7 min. The 16S rRNA gene sequencing was carried by the ABI PRISM 3100^®^ Genetic Analyzer (Applied Bioscience and Hitachi, Foster City, CA, USA). The sequences were quality checked, and low-quality regions were removed from the sequence ends using BioEdit software v.7.2.6.1. The quality-checked sequences of the isolates were compared to available sequences in the NCBI GenBank using the BLAST searching program with the megablast algorithm and the database nt. The sequences were aligned using the ClustalW algorithm and the phylogenetic tree of 16S rRNA sequences was created by the neighbor joining algorithm with 1000 bootstraps using MEGA v.7.0.0.

### 4.3. Preparation of Ethyl Acetate Extracts from the Culture Broths

The endophytic bacterial strains were cultured in 500 mL nutrient broth (NB, Himedia, Mumbai, India) for 7 days at 37 °C under the shaking condition at 150 rpm, and the cultures were then centrifuged at 10.000 rpm for 10 min. The cell-free supernatants were extracted with ethyl acetate (1:1 *v/v*, 5 times) overnight at room temperature, and the ethyl acetate extractions were then evaporated under the reduced pressure for 12–24 h at 50 °C) to remove ethyl acetate and obtain the crude extracts.

### 4.4. Pharmacological Properties of Bacterial Crude Extracts

#### 4.4.1. Antimicrobial Activity

Antimicrobial activity of the bacterial extracts was tested against five reference microbes obtained from Mientrung Institute for Scientific Research, Thua Thien Hue province, Vietnam, i.e., *Staphylococcus aureus* ATCC 25923, *Enterococcus faecalis* ATCC 29212, *Escherichia coli* ATCC 25922, *Pseudomonas aegurinosa* ATCC 27853, and *Candida albicans* ATCC 10231.

Minimum inhibitory concentrations (MICs) of the extracts against the reference microorganisms were determined using the broth microdilution method as described by Dat et al. [85]. Briefly, 100 µL of the bacterial inoculum (1 × 10^6^ CFU/mL) was added to wells containing 100 µL of the extracts at a range of different concentrations in 96-well plates. The plate was incubated at 37 °C for 24 h, the absorbance at 630 nm was then measured using ELx800 absorbance microplate reader (BioTek Instruments, Winooski, VT, USA). MICs of the antibacterial extracts were determined as the lowest concentration, at which there was no growth of the bacteria. For the yeast, 100 µL of the inoculum (2 to 5 × 10^5^ CFU/mL for yeast) was added to wells containing 100 µL of the extracts at a range of different concentrations in 96-well plates. The plate was incubated at 28 °C for 48 h. MICs of the anti-yeast extracts were determined as the lowest concentration, at which there was no growth of the yeast by the absorbance at 530 nm using an ELx800 absorbance microplate reader (BioTek Instruments, Winooski, VT, USA). The antibiotics ciprofloxacin and fluconazole were used as positive controls for the tested bacteria and yeast, respectively.

#### 4.4.2. Antioxidant Activity

The antioxidant effect of the extracts was determined by DPPH and ABTS radical scavenging assays [70].

DPPH radical scavenging effect of the extracts was determined by measuring the decrease in absorbance of DPPH radical solution in the presence of the extracts. In brief, 10 μL of extracts was added to 190 μL of DPPH (0.1 mg/mL) in 96-well plates. The solution was mixed for 1 min and incubated at room temperature for 30 min, and the absorbance of the reaction mixture was then recorded at 517 nm using an ELx800 absorbance microplate reader (BioTek Instruments, Winooski, VT, USA). Ascorbic acid was used as a positive control. The DPPH radical scavenging activity was calculated as follows: DPPH scavenging activity (%) = 100 × [Ac − (As − Asb)/Ac]. Where: Ac is the absorbance of the control (only DPPH solution), As is absorbance of sample (extract with DPPH), and Asb is the absorbance of the sample blank (extract without DPPH).

ABTS radical scavenging effect of the extracts was determined by measuring the decrease in absorbance of ABTS radical solution in the presence of the extracts. In brief, two solutions (ABTS 7 mM and potassium persulfate 2.45 mM) were mixed and allowed to stand in the dark at room temperature for 16 h before use in order to produce ABTS radical solution. The ABTS radical solution was then diluted with ethanol to give an absorbance of 0.700 ± 0.02 at 734 nm. Ten microliters of the extracts was added to 190 μL of ABTS radical solution in 96-well plates. The mixture was incubated at room temperature for 10 min, and the absorbance of the reaction was then recorded at 734 nm using ELx800 absorbance microplate reader (BioTek Instruments, Winooski, VT, USA). Ascorbic acid was used as a positive control. The ABTS radical scavenging activity was calculated as follows: ABTS scavenging activity (%) = 100 × [Ac − (As − Asb)/Ac]. Where: Ac is the absorbance of the control (only ABTS solution), As is absorbance of sample (extract with ABTS), and Asb is the absorbance of the sample blank (extract without ABTS).

#### 4.4.3. α-Amylase and α-Glucosidase Activities

The α-amylase (A8220, Sigma-Aldrich, St. Louis, MO, USA) enzyme inhibitory effect of the extracts was determined according to the described method by Dat et al. [70]. In brief, starch azure was suspended in 0.05 M Tris-HCl buffer (pH 6.9) containing 0.01 M CaCl_2_, and the substrate solution was then boiled for 5 min and pre-incubated at 37 °C for 5 min. The reaction mixture consisting of 50 µL of the extract was incubated with 50 µL of the substrate solution and 25 µL of α-amylase solution in Tris-HCl buffer (2 U/mL) in 96-well plates at 37 °C for 10 min. The reaction was stopped by adding 75 µL of acetic acid 50%, and the reaction solution was then centrifuged at 3000 rpm for 5 min at 4 °C. The absorbance of the supernatant was recorded at 650 nm using an ELx800 absorbance microplate reader (BioTek Instruments, Winooski, VT, USA). The inhibition activity was calculated as follows: inhibition (%) = 100 × [1 − (As − Abs)/(Ac − Acb)]. Where: As is the absorbance of the sample (extract with enzyme), Asb is the absorbance of the sample blank (extract without enzyme), Ac is the absorbance of the control (100% enzyme activity, only solvent with enzyme), and Acb is the absorbance of the control blank (0% enzyme activity, only solvent without enzyme). Acarbose was used as a positive control.

α-Glucosidase (G0660, Sigma-Aldrich, St. Louis, MO, USA) enzyme inhibitory effect of the extracts was determined according to the described method by Dat et al. (2021). In brief, the reaction mixture consisting of 50 μL of the extract was incubated with 100 μL of 0.1 M potassium phosphate buffer (pH 6.8) containing α-glucosidase solution (0.5 U/mL) in 96-well plates at 37 °C for 10 min. The reaction was started by adding 50 μL of 5 mM 4-Nitrophenyl β-D-glucopyranoside (pNPG), followed by incubation at 37 °C for 30 min. The absorbance of released p-nitrophenol was recorded at 405 nm using an ELx800 absorbance microplate reader (BioTek Instruments, Winooski, VT, USA). The inhibition activity was calculated as follows: inhibition (%) = 100 × [1 − (As − Asb)/(Ac − Acb)]. Where: As is the absorbance of the sample (extract with enzyme), and Asb is the absorbance of the sample blank (extract without enzyme), Ac is the absorbance of the control (100% enzyme activity, only solvent with enzyme), and Acb is the absorbance of control blank (0% enzyme activity, only solvent without enzyme). Acarbose was used as a positive control.

#### 4.4.4. Xanthine Oxidase Inhibitory Activity

The xanthine oxidase (XO) inhibitory effect of the extracts was determined according to the method described by Nguyen et al. [87] with minor modifications. In brief, the reaction mixture consisting of 50 µL of the extract, 35 µL of 70 mM phosphate buffer (pH7.5), and 30 µL of enzyme solution (0.01 units/mL in 70 mM phosphate buffer, pH 7.5) was prepared immediately before use. Subsequently, the reaction mixture was preincubated at 25 °C for 15 min, and the reaction was then initiated by adding 60 µL of substrate solution (150 mM xanthine in 70 mM phosphate buffer, pH 7.5). The reaction mixture was incubated at 25 °C for 30 min, and the reaction was then stopped by adding 25 µL of 1 N HCl. The absorbance of the mixture was measured at 290 nm using an ELx800 absorbance microplate reader (BioTek Instruments, Winooski, VT, USA). The XO inhibition activity was calculated as follows: inhibition (%) = 100 × [1 − (As − Asb)/(Ac − Acb)]. Where: As is the absorbance of the sample (extract with enzyme), Asb is the absorbance of the sample blank (extract without enzyme), Ac is the absorbance of the control (100% enzyme activity, only solvent with enzyme), and Acb is the absorbance of the control blank (0% enzyme activity, only solvent without enzyme). Allopurinol was used as a positive control.

#### 4.4.5. Cytotoxic Activity

Cytotoxic activity of the extracts against three cancer cell lines A549 (human lung carcinoma), MCF-7 (human breast carcinoma), and HeLa (human cervix carcinoma) was determined by Sulforhodamine B (SRB) assay as previously described by Skehan et al. [88]. Camptothecin was used as a positive control.

### 4.5. Gas Chromatography-Mass Spectrometry (GC-MS) Analysis

GC–MS analyses were conducted using the Agilent 7890B gas chromatograph-assisted Agilent 5977A mass detector (Agilent Technologies, Stanta Clara, CA, USA). An HP-5MS capillary column (30 m × 0.250 mm × 0.25 µM film thickness; Agilent Technologies, Stanta Clara, CA, USA) was used for separation. The samples were diluted in hexane (1:10) and volumes of 1 µL were injected (splitless mode) into the GC system at a flow rate of 1 mL/min. Helium (99.999% of purity) was the carrier gas with a flow rate of 1 mL/min. The oven temperature was set at 60 °C and held for 2 min, then increased to 260 °C at a rate of 5 °C/min and held at this temperature for 1 min. The inlet temperature was 260 °C and the ionization source temperature was 280 °C. The solvent delay was 3.00 min. The MS detector was operated in the EI mode at 70 eV, in the range of *m*/*z* 50–550, full scan mode. Data handling was performed using the Agilent ChemStation software C.01.10 (Agilent Technologies, Stanta Clara, CA, USA). The compounds were identified by comparing the spectra with a stored MS library (W8N08 and NIST08) with minimum matching quality of 90%. The relative percent of individual components was calculated based on GC peak areas.

### 4.6. Genome Sequencing, Assembly and Annotation of Biosynthesis Gene Clusters of Secondary Metabolites

The endophytic bacterial strains were cultured in nutrient broth (NB, Himedia, Mumbai, India) overnight at 37 °C under the shaking condition at 150 rpm. Cells were harvested by centrifugation at 10,000× *g* for 10 min, and genomic DNA was isolated using the QIAamp DNA Mini Kit according to the manufacturer’s protocol (QIAGEN, Hilden, Germany). The genome of the bacterial strains was sequenced by PacBio Sequel technology (PacBio, Menlo Park, CA, USA) according to the manufacturer’s instructions.

The PacBio reads were assembled using HGAP4 (SMRT Link v.6.0.0.47841) [89]. The completeness of the genome assembly was estimated by Quality Assessment Tool for Genome Assemblies (QUAST v4.6.3; http://bioinf.spbau.ru/quast/; accessed on 5 September 2021) [90] and Benchmarking Universal Single-Copy Orthologs (BUSCO v5.2.2, https://busco.ezlab.org/; accessed on 5 September 2021) [91]. Gene prediction for the complete genome sequence was performed using Prokka (v1.13) [92]. Genes involved in secondary metabolic pathways were predicted using secondary metabolite analysis shell (antiSMASH bacterial version v5.1.2, http://antismash.secondarymetabolites.org/; accessed on 10 September 2021) [93]. The predicted biosynthetic gene clusters were also searched for previously known biosynthetic pathways using the Natural Product Domain Seeker (NaPdoS) (http://napdos.ucsd.edu/; accessed on 10 September 2021) [94]. Biosynthetic gene cluster similarity networks and gene cluster families were generated using BiG-SCAPE (https://bigscape-corason.secondarymetabolites.org/; accessed on 10 September 2021) with default settings [95].

## 5. Conclusions

In the present study, twenty-three endophytic bacteria belonging to nine genera *Streptomyces*, *Bacillus*, *Pseudovibrio*, *Microbacterium*, *Brevibacterium*, *Microbulbifer*, *Micrococcus*, *Rossellomorea*, and *Paracoccus* were isolated from the fresh roots of the mangrove plant *R. apiculata*. The ethyl acetate extracts of the endophytic bacteria were evaluated for their pharmacological properties including antimicrobial, antioxidant, α-amylase and α-glucosidase inhibitory, xanthine oxidase inhibitory and cytotoxic activities. GC-MS analyses identified major volatile organic compounds from the ethyl acetate extract of the most potential strains *Bacillus* sp. RAR_GA_16, *Rossellomorea vietnamensis* RAR_WA_32, and *Bacillus* sp. RAR_M1_44. Genome studies found gene clusters related to the biosynthesis of secondary metabolites from the bacteria endophytes. Further investigations of the high bioactive strains—such as fermentation and isolation of pure bioactive compounds, and heterologous expression of novel BGCs in appropriate expression hosts—may allow exploring and exploiting the promising bioactive compounds for future drug development.

## Figures and Tables

**Table 1:** Identification of the endophytic bacterial strains by 16S rRNA gene sequences.

Strain ID	Identification by 16S rRNA Gene Sequence	NCBI BLAST Identity (%)	Accession Number
RAR_GA_12	*Streptomyces* sp.	99.8	MT229086
RAR_GA_16	*Bacillus* sp.	100	OK649239
RAR_WA_18	*Bacillus* sp.	99.8	MT229092
RAR_M1_23	*Pseudovibrio* sp.	99.7	MT229094
RAR_M1_26	*Bacillus* sp.	99.6	MT229093
RAR_GA_31	*Microbacterium* sp.	99.9	MT229099
RAR_WA_32	*Rossellomorea vietnamensis*	100	OK649240
RAR_M1_36	*Brevibacterium* sp.	99.9	MT229096
RAR_M1_41	*Microbulbifer* sp.	99.8	MT229085
RAR_GA_42	*Micrococcus* sp.	99.9	MT229088
RAR_M1_44	*Bacillus* sp.	100	OK649238
RAR_M1_45	*Bacillus* sp.	99.9	MT229100
RAR_M1_49	*Paracoccus* sp.	99.9	MT229090
RAR_WA_50	*Bacillus* sp.	99.8	MT229104
RAR_M1_53	*Streptomyces* sp.	99.9	MT229089
RAR_M1_54	*Streptomyces* sp.	100	MT229103
RAR_GA_57	*Streptomyces* sp.	99.9	MT229098
RAR_M1_58	*Bacillus velezensis*	100	MT229097
RAR_M1_60	*Bacillus* sp.	99.9	MT229102
RAR_M1_61	*Bacillus velezensis*	100	MT229087
RAR_GA_63	*Streptomyces* sp.	99.7	MT229095
RAR_GA_64	*Streptomyces* sp.	100	MT229101
RAR_M1_66	*Microbacterium* sp.	99.8	MT229091

**Table 2:** Antimicrobial activity of the bacterial extracts (MIC, µg/mL).

Strain ID	*S. aureus*	*E. faecalis*	*E. coli*	*P. aegurinosa*	*C. albicans*
RAR_GA_12	64	128	-	-	64
RAR_GA_16	16	32	256	-	32
RAR_WA_18	-	64	128	256	256
RAR_M1_23	128	-	-	-	-
RAR_M1_26	-	-	-	-	-
RAR_GA_31	-	-	-	64	128
RAR_WA_32	-	-	32	64	128
RAR_M1_36	64	32	128	-	-
RAR_M1_41	-	-	-	-	
RAR_GA_42	-	-	-	-	-
RAR_M1_44	32	64	128	-	64
RAR_M1_45	-	128	128	256	-
RAR_M1_49	-	-	-	-	-
RAR_WA_50	32	-	-	64	64
RAR_M1_53	-	64	-	256	-
RAR_M1_54	-	128	-	-	-
RAR_GA_57	-	32	-	16	64
RAR_M1_58	-	256	-	-	
RAR_M1_60	128	-	128	-	32
RAR_M1_61	-	-	-	-	-
RAR_GA_63	64	256	-	-	
RAR_GA_64	-	-	64		-
RAR_M1_66	128	-	-	64	128
Ciprofloxacin	1	2	0.5	0.5	-
Fluconazole					2

**Table 3:** DPPH and ABTS radical scavenging activity of the bacterial extracts (IC_50_, µg/mL).

Strain ID	DPPH Radical Scavenging Activity	ABTS Radical Scavenging Activity
RAR_GA_12	>100	>100
RAR_GA_16	72.24 ± 5.26	56.29 ± 4.61
RAR_WA_18	>100	>100
RAR_M1_23	61.23 ± 3.26	76.32 ± 3.51
RAR_M1_26	>100	>100
RAR_GA_31	72.36 ± 3.95	>100
RAR_WA_32	43.52 ± 3.87	66.43 ± 5.25
RAR_M1_36	44.34 ± 2.76	64.51 ± 3.26
RAR_M1_41	>100	>100
RAR_GA_42	51.64 ± 2.68	76.29 ± 3.75
RAR_M1_44	81.34 ± 6.18	77.35 ± 5.82
RAR_M1_45	>100	>100
RAR_M1_49	>100	>100
RAR_WA_50	>100	81.45 ± 3.64
RAR_M1_53	>100	>100
RAR_M1_54	>100	>100
RAR_GA_57	>100	59.43 ± 2.68
RAR_M1_58	88.32 ± 4.13	67.48 ± 2.46
RAR_M1_60	>100	>100
RAR_M1_61	65.23 ± 3.23	77.21 ± 3.24
RAR_GA_63	>100	>100
RAR_GA_64	>100	>100
RAR_M1_66	>100	71.35 ± 3.57
Ascorbic acid	29.14 ± 4.27	26.35 ± 3.24

**Table 4:** α-Amylase, α-glucosidase, and xanthine oxidase inhibitory activities of the bacterial extracts (IC_50_, µg/mL).

Strain ID	α-Amylase Inhibition	α-Glucosidase Inhibition	XO Inhibition
RAR_GA_12	>200	74.26 ± 4.12	>100
RAR_GA_16	73.27 ± 3.45	85.73 ± 5.26	94.36 ± 4.74
RAR_WA_18	>200	>200	54.57 ± 2.53
RAR_M1_23	131.36 ± 5.41	>200	>100
RAR_M1_26	>200	73.64 ± 3.78	>100
RAR_GA_31	64.42 ± 3.35	81.32 ± 4.42	>100
RAR_WA_32	33.51 ± 4.62	53.68 ± 3.12	74.38 ± 3.81
RAR_M1_36	51.67 ± 2.98	>200	75.36 ± 3.76
RAR_M1_41	>200	0>200	>100
RAR_GA_42	>200	>200	82.35 ± 4.13
RAR_M1_44	86.38 ± 5.39	94.14 ± 5.28	64.43 ± 4.56
RAR_M1_45	>200	>200	>100
RAR_M1_49	58.34 ± 2.47	55.16 ± 4.39	>100
RAR_WA_50	77.64 ± 3.68	>200	68.46 ± 3.47
RAR_M1_53	>200	>200	>100
RAR_M1_54	>200	>200	74.38 ± 3.81
RAR_GA_57	83.45 ± 4.25	57.38 ± 3.42	>100
RAR_M1_58	68.34 ± 3.42	>200	>100
RAR_M1_60	>200	>200	71.44 ± 3.67
RAR_M1_61	>200	193.44 ± 6.73	>100
RAR_GA_63	128.34 ± 6.32	>200	>100
RAR_GA_64	>200	>200	55.29 ± 3.27
RAR_M1_66	>200	>200	>100
Acarbose	89.34 ± 3.61	217.46 ± 6.38	-
Allopurinol	-	-	6.12 ± 0.61

**Table 5:** Cytotoxic activity of bacterial extracts against cancer cell lines (IC_50_, µg/mL).

Strain ID	MCF-7	A549	Hela
RAR_GA_12	>100	>100	>100
RAR_GA_16	36.48 ± 2.63	89.53 ± 5.31	41.27 ± 3.42
RAR_WA_18	> 100	>100	>100
RAR_M1_23	>100	>100	>100
RAR_M1_26	>100	>100	>100
RAR_GA_31	>100	>100	>100
RAR_WA_32	>100	21.52 ± 3.22	57.67 ± 4.75
RAR_M1_36	83.24 ± 4.51	>100	>100
RAR_M1_41	>100	>100	>100
RAR_GA_42	53.17 ± 2.84	>100	>100
RAR_M1_44	61.32 ± 3.22	51.32 ± 4.21	97.53 ± 5.31
RAR_M1_45	>100	>100	>100
RAR_M1_49	>100	>100	>100
RAR_WA_50	61.32 ± 3.22	36.48 ± 2.32	>100
RAR_M1_53	>100	>100	>100
RAR_M1_54	>100	>100	>100
RAR_GA_57	77.36 ± 2.31	>100	>100
RAR_M1_58	>100	>100	>100
RAR_M1_60	>100	>100	>100
RAR_M1_61	>100	>100	>100
RAR_GA_63	>100	>100	>100
RAR_GA_64	>100	>100	>100
RAR_M1_66	>100	>100	>100
Camptothecin	4.75 ± 0.41	2.47 ± 0.36	3.56 ± 0.62

**Table 6:** The volatile chemical composition of the bacterial extracts.

No.	Composition	RT/min	Matching Quality	Quantity (%)	Biological Activity	Refs.
*Bacillus* sp. RAR_GA_16						
1	Hexahydro-pyrrolo[1,2-a]pyrazine-1,4-dione	18.830	96	13.15	Antimicrobial, antioxidant	[28,29]
2	3-Isobutylhexahydropyrrolo[1,2-a]pyrazine-1,4-dione	21.125	91	8.15	Antimicrobial, nematicidal, and anti-mutagenic	[30,31,32]
3	Palmitic acid	21.413	98	2.09	Antibacterial	[33]
4	Diisooctyl phthalate	22.022	95	6.90	Antibacterial, antifungal	[34,35]
5	Linoleic acid	23.454	99	1.57	Antibacterial	[33,36]
6	9(*E*)-Octadecenoic acid	23.517	99	9.17	Antibacterial	[37]
7	Stearic acid	23.763	98	3.02	Antibacterial	[33]
*Rossellomorea vietnamensis* RAR_WA_32						
1	Benzyl alcohol	8.045	96	0.12	Antibacterial	[38,39]
2	2,3,5,6-Tetramethylpyrazine	9.494	91	0.16	Antioxidant, anticancer, anti-inflammatory	[40,41,42]
3	Phenylethyl alcohol	10.225	97	0.16	Antibacterial	[43,44]
4	Benzoic acid	11.783	94	0.25	Antimicrobial	[45]
5	Methyl phenylacetate	12.022	94	0.19	Antimicrobial	[46,47]
6	Benzeneacetic acid	14.193	94	1.92	Antimicrobial	[48,49]
7	Benzenepropanoic acid	16.367	96	0.38	Antimicrobial	[50]
8	Docosane	20.327	91	0.09	-	-
9	2,4-Di-tert-butylphenol	20.775	97	0.3	Antifungal, antioxidant	[51,52]
10	Hexahydropyrrolo[1,2-a]pyrazine-1,4-dione	26.144	96	0.38	Antibacterial, antioxidant	[28,29]
11	Myristic acid	26.351	94	0.08	Antibacterial	[53,54]
12	Methyl 13-methylmyristate	26.911	97	0.12	-	-
13	12-Methyltetradecanoic acid	27.078	94	0.23	Antifungal, anticancer	[55,56]
14	Pentadecanoic acid	27.717	98	0.64	-	-
15	Methyl palmitate	29.702	95	0.76	Antibacterial, anti-inflammatory	[57,58]
16	3-Isobutylhexahydropyrrolo[1,2-a]pyrazine-1,4-dione	29.836	95	4.56	Antimicrobial, nematicidal, and anti-mutagenic	[30,31,32]
17	Palmitic acid	30.482	99	5.56	Antibacterial	[33]
18	Methyl 14-methylhexadecanoate	31.084	92	0.18	-	-
19	11-Octadecenoic methyl ester	33.090	95	0.08	-	-
20	Methyl stearate	33.458	96	0.14	-	-
21	9-Octadecenoic acid	33.755	98	0.79	Antibacterial	[59]
22	Stearic acid	34.135	99	3.55	Antibacterial	[33]
23	1,3,3a,6,7,9a-Hexahydro-cis-cycloocta[c]furan	35.701	90	0.23	-	-
24	Cyclo(phe-pro)	36.941	90	0.15	Antimicrobial, anticancer	[60,61,62]
25	3-Benzylhexahydropyrrolo[1,2-a]pyrazine-1,4-dione	37.704	95	1.87	Anti-biofilm, anti-quorum sensing	[63]
26	Dioctyl phthalate	40.507	91	58.09	Antibacterial, tyrosinase inhibitory	[64,65,66]
*Bacillus* sp. RAR_M1_44						
1	Palmitic acid	21.416	97	0.51	Antimicrobial, antioxidant	[33]
2	(Z,Z)-9,12-Octadecadienoic acid	23.459	99	0.33	Anti-inflammatory, nematicidal, and hepatoprotective	[67]
3	cis-Vaccenic acid	23.521	99	1.93	Antibacterial, hypolipidemic	[68]
4	Stearic acid	23.764	99	0.34	Antibacterial, antifungal	[33]
5	1,2-Benzenedicarboxylic acid	23.978	90	0.99	Antifungal, anticancer	[33,36]

Compounds with minimum match quality of 90%.

**Table 7:** Genomic features of the bacterial endophytes.

Genomic Features	RAR_GA_16	RAR_WA_32	RAR_M1_44
Size of the genome assembly (bp)	4,394,636	4,494,267	3,768,026
GC content (%)	40.69	44.09	41.38
Contigs	22	4	5
Maximum Contig Length (bp)	770,470	2,474,194	2,582,281
N50 Contig Length (bp)	501,856	2,474,194	2,582,281
CDS	4610	4529	3807
rRNA	27	22	24
tRNA	89	111	81
tmRNA	1	0	1
Complete genome (%)	96.6	99.5	99.8

**Table 8:** Predicted biosynthesis gene clusters of secondary metabolites from the bacterial genomes.

Cluster	Length	Types	Most Similar Known Cluster	MIBiG BGC-ID	Similarity
*Bacillus* sp. RAR_GA_16					
Cluster 1	21,787	LAP, bacteriocin	-	-	-
Cluster 2	14,207	Lassopeptide	Paeninodin	BGC0001356	80%
Cluster 3	28,959	Siderophore	-	-	-
Cluster 4	37,598	T3PKS	-	-	-
Cluster 5	21,821	Terpene	Butirosin A/butirosin B	BGC0000693	7%
Cluster 6	20,837	Terpene	Carotenoid	BGC0000645	50%
Cluster 7	24,479	-	S-layer glycan	BGC0000796	14%
Cluster 8	15,240	-	Thaxteramide C	BGC0002025	7%
Cluster 9	4858	-	Fengycin	BGC0001095	20%
Cluster 10	8288	-	Capsular polysaccharide	BGC0000758	4%
Cluster 11	11,638	-	-	-	-
Cluster 12	6299	-	-	-	-
Cluster 13	3468	-	-	-	-
Cluster 14	15,194	-	-	-	-
Cluster 15	9297	-	-	-	-
Cluster 16	15,779	-	-	-	-
Cluster 17	11,263	-	-	-	-
Cluster 18	9795	-	-	-	-
Cluster 19	9448	-	-	-	-
Cluster 20	16,830	-	-	-	-
Cluster 21	7027	-	-	-	-
Cluster 22	5280	-	-	-	-
*Rossellomorea vietnamensis* RAR_WA_32					
Cluster 1	21,869	Terpene	Pyxidicycline A/pyxidicycline B	BGC0001940	6%
Cluster 2	20,443	Saccharide	A40926	BGC0000289	3%
Cluster 3	22,620	Saccharide	Carotenoid	BGC0000645	33%
Cluster 4	121,332	Saccharide	S-layer glycan	BGC0000796	14%
Cluster 5	50,793	Fatty_acid, saccharide, terpene	Carotenoid	BGC0000645	50%
Cluster 6	19,105	LAP, RiPP-like	-	-	-
Cluster 7	20,392	Saccharide	-	-	-
Cluster 8	70,095	Saccharide,T3PKS	-	-	-
Cluster 9	29,497	Saccharide	-	-	-
Cluster 10	21,005	Fatty_acid	-	-	-
Cluster 11	30,306	Saccharide	-	-	-
Cluster 12	34,473	Saccharide	-	-	-
Cluster 13	21,368	Saccharide	-	-	-
Cluster 14	24,846	Saccharide	-	-	-
Cluster 15	30,807	Saccharide	-	-	-
Cluster 16	35,649	Saccharide	-	-	-
*Bacillus* sp. RAR_M1_44					
Cluster 1	28,756	Siderophore, terpene	Carotenoid	BGC0000645	50%
Cluster 2	28,411	Betalactone	Fengycin	BGC0001095	53%
Cluster 3	83,725	NRPS	Lichenysin	BGC0000868	85%
Cluster 4	8013	Other	Teichuronic acid	BGC0001184	75%
Cluster 5	41,421	Other	Bacilysin	BGC0000381	85%
Cluster 6	21,877	Terpene	-	-	-
Cluster 7	41,097	T3PKS	-	-	-
Cluster 8	26,707	Bacteriocin			
Cluster 9	32,416	Betalactone			
Cluster 10	15,922	-	-	-	-
Cluster 11	5255	-	-	-	-
Cluster 12	13,700	-	-	-	-
Cluster 13	8838	-	-	-	-
Cluster 14	16,010	-	-	-	-
Cluster 15	3876	-	-	-	-
Cluster 16	9767	-	-	-	-

**Table 9:** Predicted pathway products of KS and C domains from the bacterial genomes.

KS/C Domain ID	Database Match ID	Percent Identity (%)	E-Value	Pathway Product
*Bacillus* sp. RAR_GA_16				
KS1	AmphI_Q93NX9_1mod	42	4e-7	Amphotericin
KS2	SpnB_Q9ALM5_1KSB	35	2e-7	Spinosad
KS3	FabF_Bacillus_FAS	76	0	Fatty acid
KS4	DynE_AAN79725_ene10	45	9e-6	Dynemicin
*Rossellomorea vietnamensis* RAR_WA_32				
KS1	KirAI_CAN89631_2T	40	5e-7	Kirromycin
KS2	FabB_Streptomyces_FAS	41	3e-6	Fatty acid
KS3	MycAIII_Q83WE8_2KSB	44	1e-6	Mycinamicin
KS4	FabF_Bacillus_FAS	71	0.0	Fatty acid
KS5	RifB_O52545_1mod	26	1e-6	Rifamycin
*Bacillus* sp. RAR_M1_44				
KS1	Myca_YP881572_1KSB	40	2e-6	Mycocerosic acid
KS2	FabF_Bacillus_FAS	48	1e-108	Fatty acid
KS3	FabF_Bacillus_FAS	84	0.0	Fatty acid
C1	Liche1_C1_start	51	3e-135	Lychenicin
C2	Liche1_C1_start	24	4e-25	Lychenicin
C3	Liche1_C1_start	22	6e-18	Lychenicin
C4	Surfa5_C1_DCL	57	5e-151	Surfactin
C5	Surfa5_C1_DCL	24	2e-24	Surfactin
C6	Surfa5_C1_DCL	22	3e-12	Surfactin
C7	Surfa5_C1_DCL	24	3e-8	Surfactin
C8	Liche3_C1_DCL	58	5e-151	Lychenicin
C9	Act3_C3_LCL	39	6e-86	Actinomycin
C10	Act3_C3_LCL	23	2e-20	Actinomycin
C11	Liche1_C3_LCL	38	2e-77	Lychenicin

## Data Availability

The 16S rRNA gene sequences of isolates are available in the NCBI database under accession numbers: MT229085-MT229104, OK649238-OK649240. The whole genome shotgun projects have been deposited at DDBJ/ENA/GenBank under the accession numbers: JAIUKX000000000-JAIUKZ000000000. The versions described in this paper are versions JAIUKX010000000-JAIUKZ010000000.

## Supplementary Materials

The following are available online at https://www.mdpi.com/article/10.3390/antibiotics10121491/s1, Table S1. Comparison of the GC-MS peaks of the extract of *Bacillus* sp. RAR_GA_16 with the spectra library. Table S2. Comparison of the GC-MS peaks of the extract of *R. vietnamensis* RAR_WA_32 with the spectra library. Table S3. Comparison of the GC-MS peaks of the extract of *Bacillus* sp. RAR_M1_44 with the spectra library.
