# Supplementary material for: Pharmacological Properties, Volatile Organic Compounds, and Genome Sequences of Bacterial Endophytes from the Mangrove Plant Rhizophora apiculata Blume
Source: Antibiotics (Basel). 2021 Dec 5;10(12):1491. doi: 10.3390/antibiotics10121491 (PMC8698355; doi:10.3390/antibiotics10121491)
Supplement: Supplementary file 1 [file antibiotics-10-01491-s001.zip › antibiotics-1485786-supplementary.pdf]

Table S1. Comparison of the GC-MS peaks of the extract of Bacillus sp. RAR_GA_16 with the spectra library.

Peak	Compound	RT min	Qual	% of Total
1	(4Z)-4-Heptenal	4.148	43	6.99%
2	Butanoic acid, 4-methylpentyl ester	5.269	27	1.63%
3	Palmitic acid	7.097	47	1.08%
4	3-Methyl-N-[(E)-3-methylbutylidene]-1-butanamine	7.266	72	1.84%
5	2,3-Dihydro-3,5-dihydroxy-6-methyl-4h-pyran-4-one	9.126	72	1.49%
6	5-Methyl-1,2-hexadiene	9.895	53	1.53%
7	1-Butyl-1H-pyrrole	10.063	59	0.92%
8	3-Methyl-1-pentene	10.476	46	0.89%
9	2-Thiopheneacetic acid, phenyl ester	10.638	25	1.01%
10	Benzeneacetic acid	10.978	60	0.97%
11	3-Pyrrolidin-2-yl-propionic acid	18.248	40	2.45%
12	Pyrrolo[1,2-a]pyrazine-1,4-dione, hexahydro-	18.83	96	13.15%
13	3,5-Dimethoxyphenol	19.679	35	4.21%
14	2,2,4,4-Tetramethyl-6-oxabicyclo[3.1.0]hexan-3-one	19.992	43	0.74%
15	3-Isobutylhexahydropyrrolo[1,2-a]pyrazine-1,4-dione	20.912	72	3.27%
16	3-Isobutylhexahydropyrrolo[1,2-a]pyrazine-1,4-dione	21.125	91	8.15%
17	3-Isobutylhexahydropyrrolo[1,2-a]pyrazine-1,4-dione	21.229	64	2.16%
18	3,9-Dihydroxy-1,7-diazatricyclo[7.3.0.03,7]dodecane-2,8-dione	21.277	60	1.46%
19	2,2-Dimethylcyclobutanone	21.317	25	2.38%
20	Palmitic acid	21.413	98	2.09%
21	Diisooctyl phthalate	22.022	95	6.90%
22	Linoleic acid	23.454	99	1.57%
23	9(E)-Octadecenoic acid	23.517	99	9.17%
24	Cyclododecane	23.694	59	2.51%
25	Stearic acid	23.763	98	3.02%
26	cis-2-Thioxo-5,6-trimethylene-2,3,5,6-tetrahydropyrimidin-4(1H)-one	23.86	35	9.80%
27	6-Dodecanol acetate	23.932	25	4.44%
28	5,8-Dimethyl-[1,2,4]triazolo[4,3-a]pyrazin-3-amine	24.194	38	2.52%
29	3-Benzylhexahydropyrrolo[1,2-a]pyrazine-1,4-dione	26.195	64	1.67%

Table S2. Comparison of the GC-MS peaks of the extract of R. vietnamensis RAR_WA_32 with the spectra library.

Peak	Compound	RT min	Qual	% of Total
1	3-Methylbutyric acid	3.727	78	0.71%
2	2-Methylbutyric acid	3.911	83	0.74%
3	3-Methyl-1,3-oxazolidin-2-one	4.313	50	0.24%
4	Sulfide, hexyl vinyl	4.419	42	0.22%
5	1-Ethyl-1-methyl-2-oxohydrazine	5.386	47	0.11%
6	1-Nonene	5.798	46	0.13%
7	1,2,3,4,5-Pentamethoxycyclopentane	6.478	43	0.11%
8	1,2,3,4,5-Pentamethoxycyclopentane	6.558	43	0.11%
9	Isobutyl isothiocyanate	7.098	78	0.08%
10	Benzyl alcohol	8.045	96	0.12%
11	Nanofin	8.101	50	0.09%
12	2-Isopropyl-1,3-dimethylaziridine	8.338	50	0.10%
13	2,3,5,6-Tetramethylpyrazine	9.494	91	0.16%
14	Monomethyl succinate	9.949	86	0.13%
15	Phenylethyl alcohol	10.225	97	0.16%
16	2,2-Dimethyl-1,3-propanediol	10.605	47	0.20%
17	Benzoic acid	11.783	94	0.25%
18	Methyl phenylacetate	12.022	94	0.20%
19	2-Piperidinone	12.217	83	0.15%
20	Benzeneacetic acid	14.193	94	1.92%
21	Benzenepropanoic acid	16.367	96	0.38%
22	Docosane	20.327	91	0.09%
23	2,4-Di-tert-butylphenol	20.775	97	0.30%
24	Decahydro-1,4-methanobenzocyclodecene	23.472	83	0.13%
25	Diisopropylethylamine	24.929	30	0.18%
26	Tetratriacontane	25.235	72	0.29%
27	Pentadecanoic acid	25.563	50	0.09%
28	2-(Dimethylhydrazono)butanal	25.751	86	0.17%
29	2-(Dimethylhydrazono)butanal	25.887	78	0.11%
30	Hexahydropyrrolo[1,2-a]pyrazine-1,4-dione	26.144	96	0.38%
31	Myristic acid	26.351	94	0.08%
32	Methyl 13-methylmyristate	26.911	97	0.12%
33	12-Methyltetradecanoic acid	27.078	94	0.23%
34	3-Isobutylhexahydropyrrolo[1,2-a]pyrazine-1,4-dione	27.491	59	1.16%
35	Pentadecanoic acid	27.717	98	0.64%
36	Pentadecanoic acid	27.906	64	2.31%
37	4-Decanone	28.162	49	0.59%
38	Pentadecanoic acid	28.44	96	0.14%
39	Methyl 14-methylpentadecanoate	28.961	91	0.16%
40	3-Isobutylhexahydropyrrolo[1,2-a]pyrazine-1,4-dione	29.456	91	1.95%
41	Methyl palmitate	29.702	95	0.76%
42	3-Isobutylhexahydropyrrolo[1,2-a]pyrazine-1,4-dione	29.836	95	4.56%
43	3-Cyclopentylpropionic acid, 3-methylbutyl ester	30.024	35	1.57%
44	Palmitic acid	30.482	99	5.56%
45	Tetradecanoic acid, 12-methyl-, methyl ester	30.918	70	0.16%
46	Methyl 14-methylhexadecanoate	31.084	92	0.18%
47	9-Octadecenoic acid	31.6	94	0.26%
48	Cyclohexylmethyl benzoate	31.771	83	1.78%

49	Trichloroacetic acid, tridecyl ester	32.035	64	0.36%
50	2,6,10,14-Tetramethylpentadecane	32.197	46	0.15%
51	1,54-Dibromotetrapentacontane	32.262	87	0.18%
52	11-Octadecenoic methyl ester	33.09	95	0.08%
53	Pyrrolidine, 1-(3A,4,7,7A-tetrahydro-1H-inden-2-yl)-, cis-	33.267	49	0.09%
54	Methyl stearate	33.458	96	0.14%
55	Icosan	33.641	86	0.16%
56	9-Octadecenoic acid	33.755	98	0.79%
57	2-Indanylacetic acid	33.988	47	0.12%
58	Stearic acid	34.135	99	3.55%
59	2,4-Methano-1H-indene, 1-chlorooctahydro-	34.391	50	0.60%
60	2-(Hydroxymethyl)norbornadiene	34.735	55	0.16%
61	1,3,3A,6,7,9A-Hexahydro-cis-cycloocta[c]furan	34.812	80	0.14%
62	Tricyclo[5.2.1.0^{2},^{6}]decan-8-ol	35.319	50	0.10%
63	1,3,3a,6,7,9a-Hexahydro-cis-cycloocta[c]furan	35.701	90	0.23%
64	Cyclo(phe-pro)	36.941	90	0.15%
65	3-Benzyl-6-isopropyl-2,5-piperazinedione	37.207	59	0.23%
66	1-Chlorohexadecane	37.275	35	0.16%
67	1-Octadecanethiol	37.555	53	0.51%
68	3-Benzylhexahydropyrrolo[1,2-a]pyrazine-1,4-dione	37.704	95	1.87%
69	5-Ethyl-1-nonene	38.281	42	0.18%
70	15-Hydroxypentadecanoic acid	39.21	56	0.13%
71	Icosane	39.333	59	0.09%
72	2-Hexadecanoyl glycerol	39.815	74	0.49%
73	Dioctyl phthalate	40.507	91	58.09%
74	Di-n-octyl phthalate	40.779	43	0.25%
75	1-Octadecene	40.951	89	0.12%
76	9-(2',2'-Dimethylpropanoilhydrazono)-3,6-dichloro-2,7-bis-[2-(diethylamino)-ethoxy]fluorene	41.022	38	0.14%
77	2,4-Diamino-6-oxo-1,6-dihydro-5-pyrimidinylformamide	41.484	55	0.29%
78	1-Butyl-2-methylcyclopropane	42.635	38	0.25%

Table S3. Comparison of the GC-MS peaks of the extract of *Bacillus* sp. RAR_M1_44 with the spectra library.

Peak	Compound	RT min	Qual	% of Total
1	dl-Glyceraldehyde	3.422	64	12.81%
2	Diglycolic acid	3.741	64	1.05%
3	2-Furanmethanol	3.913	60	1.94%
4	Thiophene, tetrahydro-3-methyl	4.249	42	3.41%
5	Triethylphosphine	4.447	23	10.65%
6	2,4-Dihydroxy-2,5-dimethyl-3(2H)-furan-3-one	6.037	35	0.62%
7	Propionic acid	6.105	38	0.95%
8	N,N'-Dimethylpiperazine	7.134	64	0.29%
9	1,1-dimethyl-2-oxohydrazine	7.336	50	0.27%
10	Thymine	7.878	86	3.12%
11	(5Z)-3-Methyl-1,5-heptadiene	8.016	17	1.00%
12	1,1-dimethyl-2-oxohydrazine	8.968	72	0.62%
13	3,5-Dihydroxy-6-methyl-2,3-dihydro-4H-pyran-4-one	9.136	59	4.30%
14	N-Acetyl-d-galactosamine	9.361	25	6.18%
15	2-Hydroxyethyl butyl sulfide	9.407	23	0.87%
16	2(R),3(S)-1,2,3,4-Butanetetrol	9.445	25	4.41%
17	n-Propylcyclopropanemethylamine	9.699	53	0.71%
18	2(3H)-Furanone, 5-butyldihydro-	9.951	64	0.42%
19	Octahydro-2,2'-Bi-2H-pyran	10.423	64	1.27%
20	2-Pentenoic acid, 3-methyl-, methyl ester, (E)-	10.494	47	1.52%
21	2-Furancarboxaldehyde, 5-(hydroxymethyl)-	10.647	87	4.23%
22	Ethanamine, 2,2-diethoxy-	10.911	47	2.57%
23	2-Methyl-2-(1-methylethyl)-1,3-oxathiolane	10.971	27	0.33%
24	D-Mannoheptulose	11.43	22	0.30%
25	trans--4-Nonene	11.656	38	2.66%
26	5(4H)-Oxazolone, 2-(1,1-dimethylethyl)-	12.204	27	0.64%
27	Allylthiourea	12.277	43	0.62%
28	Hexyl n-valerate	12.628	50	1.25%
29	2,3-Epoxyhexanol	13.049	43	0.57%
30	2-Methyl-1,3-dioxacycloheptane	13.28	49	0.70%
31	Lactone G	13.833	64	2.05%
32	4-Hydroxyphenethyl alcohol	14.01	70	0.36%
33	2,2-Dideutero-trans-1,3-dihydroxy-cyclopentane	14.381	38	15.72%
34	4-O-Hexopyranosylhexose	14.928	46	0.09%
35	3-Deoxy-d-mannoic lactone	16.68	59	4.89%
36	N-Methyl-D-aspartic acid	16.815	22	0.30%
37	Gamma-gulonolactone	17.095	35	2.21%
38	Palmitic acid	21.416	97	0.51%
39	(Z,Z)-9,12-Octadecadienoic acid	23.459	99	0.33%
40	cis-Vaccenic acid	23.521	99	1.93%
41	Stearic acid	23.764	99	0.34%
42	1,2-Benzenedicarboxylic acid	23.978	90	0.99%
